# Differential correlation patterns of serum prolactin and total antioxidant status in obstructive sleep apnea: A cross-sectional study

**DOI:** 10.1097/MD.0000000000050808

**Published:** 2026-09-25

**Authors:** Hamza Ogun, Esat Hayat, Nuran Gokbulut, Merve Yuzbasioglu, Abdullah Kansu, Ömer Faruk Özer, Deniz Kara, Fatmanur Okyaltirik

**Affiliations:** a Department of Chest Diseases, Bezmialem Vakif University, Istanbul, Turkey; b Department of Chest Diseases, Başakşehir Çam ve Sakura City Hospital, Istanbul, Turkey; c Department of Family Medicine, Şişli Hamidiye Etfal Training and Research Hospital, Istanbul, Turkey; d Department of Chest Diseases, Medipol University Faculty of Medicine, Istanbul, Turkey; e Department of Biochemistry, Bezmialem Vakif University, Istanbul, Turkey; f Department of Anesthesiology and Reanimation, Bezmialem Vakif University, Istanbul, Turkey.

**Keywords:** apnea-hypopnea duration, biomarkers, hypoxemia, obstructive sleep apnea, oxidative stress, prolactin, total antioxidant status

## Abstract

Obstructive sleep apnea (OSA) severity classification relies primarily on the Apnea-Hypopnea Index (AHI), which quantifies event frequency but does not capture event duration or hypoxemic burden. We examined whether serum prolactin and total antioxidant status (TAS) show differential correlation patterns with frequency- versus duration-based polysomnographic parameters, potentially reflecting distinct pathophysiological mechanisms. This cross-sectional study enrolled 55 participants (controls: n = 12, AHI < 5; mild/moderate OSA: n = 22, 5 ≤ AHI < 30; severe OSA: n = 21, AHI ≥ 30) undergoing overnight polysomnography. Fasting serum prolactin and TAS were measured. Spearman correlations examined biomarker associations with AHI (numerically equivalent to the Respiratory Disturbance Index in this cohort), oxygen desaturation index, total apnea-hypopnea duration, longest apnea duration, and lowest oxygen saturation; *P*-values were adjusted for multiple testing using the Benjamini–Hochberg false discovery rate procedure. Both prolactin (22.7 ± 9.8 vs 14.7 ± 7.2 vs 12.7 ± 7.3 ng/mL, *P* = .007) and TAS (1.181 ± 0.068 vs 1.122 ± 0.065 vs 1.111 ± 0.057 mmol/L, *P* = .021) decreased progressively across severity groups. TAS correlated with event frequency (AHI: *r* = −0.419, *q* = 0.013) but not with longest apnea duration (*r* = −0.223, *q* = 0.103), while prolactin correlated with both frequency (AHI: *r* = −0.360, *q* = 0.017) and duration parameters (longest apnea duration: *r* = −0.288, *q* = 0.044). However, when TAS and prolactin correlations were compared directly for each parameter using Steiger *Z* test for dependent correlations, none of the differences reached statistical significance (all *P* ≥ .65), indicating that this apparent divergence reflects differing significance thresholds rather than a formally confirmed difference in association strength. A positive correlation between prolactin and lowest oxygen saturation did not retain significance after false discovery rate correction (*q* = 0.053) and is considered exploratory. TAS and prolactin show descriptively different correlation patterns with frequency- and duration-based polysomnographic parameters; however, direct statistical comparison did not confirm these differences as significant, and the findings should be regarded as hypothesis-generating rather than confirmed evidence of distinct pathophysiological mechanisms. Larger, independent cohorts are needed to formally test whether these biomarkers diverge in their associations with OSA severity dimensions.

## 1. Introduction

Obstructive sleep apnea (OSA) affects approximately 1 billion adults globally and associates with increased cardiovascular morbidity and mortality.^[1,2]^ Current diagnosis and severity classification rely on the Apnea-Hypopnea Index (AHI), which quantifies respiratory event frequency but provides no information on event duration, oxygen desaturation depth, or sleep architecture disruption.^[3]^ This limitation is clinically significant, as AHI demonstrates substantial night-to-night variability^[4]^ and inconsistent relationships with symptoms and cardiovascular outcomes.^[5]^ These observations have prompted investigation of complementary parameters and biomarkers that might capture pathophysiologically relevant dimensions of OSA not reflected by event frequency alone.

Emerging evidence suggests apnea-hypopnea duration (AHD) represents a distinct OSA dimension. Total event duration varies substantially among patients with similar AHI values,^[6]^ and longer mean duration associates with worse hypertension outcomes.^[7]^ Paradoxically, shorter event duration predicted higher mortality in community samples,^[8]^ likely reflecting different mechanisms: prolonged events intensify acute hypoxemic stress, while frequent brief events accumulate greater sleep fragmentation. Understanding which biomarkers correlate with duration versus frequency could illuminate these distinct pathophysiological pathways.

Intermittent hypoxemia triggers oxidative stress through cyclical reactive oxygen species generation during repetitive ischemia-reperfusion cycles.^[9]^ Total antioxidant status (TAS), reflecting overall capacity to neutralize oxidative species, decreases in OSA patients and correlates inversely with AHI in some studies.^[10–12]^ However, whether oxidative stress markers correlate more strongly with event frequency (number of ischemia-reperfusion cycles) or duration (severity of individual episodes) remains unexplored.

OSA disrupts prolactin regulation through multiple potential mechanisms. Prolactin exhibits circadian variation with nocturnal elevation during sleep, particularly slow-wave sleep.^[13,14]^ Studies report variable prolactin alterations in OSA,^[15,16]^ possibly reflecting heterogeneous phenotypes or failure to account for duration parameters. Both sleep fragmentation (disrupting normal sleep-dependent secretion) and severe hypoxemia (inducing stress-mediated pituitary suppression) could alter prolactin through different pathways. Rapid eye movement (REM) sleep, characterized by upper airway muscle atonia and longer apneic events,^[17]^ demonstrates particular vulnerability and may differentially affect neuroendocrine function.

We hypothesized that serum prolactin and TAS might demonstrate differential correlation patterns with polysomnographic parameters, reflecting distinct pathophysiological mechanisms. We postulated TAS would correlate primarily with event frequency (cumulative ischemia-reperfusion burden), while prolactin might show sensitivity to both frequency and duration parameters (combined sleep fragmentation and hypoxemic severity effects). To test this, we measured both biomarkers alongside comprehensive polysomnographic assessment including frequency metrics (AHI, Respiratory Disturbance Index [RDI], and Oxygen Desaturation Index [ODI]), duration parameters (total AHD, longest apnea duration), REM sleep characteristics, and oxygenation metrics in OSA patients across severity spectrum and controls.

## 2. Materials and methods

### 2.1. Study design and setting

This cross-sectional study was conducted in the Sleep Polyclinic at the Department of Chest Diseases, Bezmialem Vakif University Hospital, Istanbul, Turkey, between February 2020 and February 2021. The study protocol was approved by the Institutional Review Board of Bezmialem Vakif University Non-Interventional Research Ethics Committee (Decision no: 24/449, Date: December 17, 2019) and conducted in accordance with the Declaration of Helsinki. All participants provided written informed consent prior to enrollment.

### 2.2. Participants

We enrolled consecutive adults aged 18 to 80 years who presented to our sleep clinic with suspected OSA based on clinical symptoms (snoring, witnessed apneas, and excessive daytime sleepiness) or screening questionnaire results. Following overnight polysomnography (PSG), participants were classified into 3 groups based on AHI according to American Academy of Sleep Medicine criteria:^[18]^ controls (AHI < 5 events/h, n = 12); mild/moderate OSA (5 ≤ AHI < 30 events/h, n = 22); and severe OSA (AHI ≥ 30 events/h, n = 21). The final study cohort comprised 55 participants.

Exclusion criteria were designed to minimize confounding effects on oxidative stress and hormonal parameters. We excluded individuals with any of the following: known cardiovascular disease (ischemic heart disease, heart failure, cerebrovascular disease, and peripheral arterial disease); metabolic disorders (diabetes mellitus and thyroid dysfunction); acute or chronic infection; inflammatory conditions (collagen tissue disease and inflammatory bowel disease); malignancy; hepatic or renal disease; hematological disorders (active bleeding, thrombosis, anemia defined as hemoglobin < 12 g/dL for women or < 13 g/dL for men); current use of medications known to affect oxidative stress or prolactin levels (antioxidant supplements, dopamine agonists/antagonists, and antipsychotics); pregnancy or lactation; or previous OSA treatment including continuous positive airway pressure (CPAP) therapy or oral appliances.

On the initial visit, all participants underwent detailed medical history, physical examination, and anthropometric measurements including height, weight, neck circumference (measured at the level of the cricothyroid membrane), and waist circumference (measured at the midpoint between the lower costal margin and iliac crest). Body mass index (BMI) was calculated as weight in kilograms divided by height in meters squared. Daytime sleepiness was assessed using the Epworth Sleepiness Scale, with scores ranging from 0 to 24.^[19]^

### 2.3. Polysomnography

All participants underwent overnight attended PSG in our sleep laboratory using a digital polysomnography system (EMBLA S4000; Remlogic) with Compumedics E series software (Compumedics Inc.). Standard montage included electroencephalography (F3-M2, F4-M1, C3-M2, C4-M1, O1-M2, and O2-M1), bilateral electrooculography, submental and bilateral anterior tibialis electromyography, electrocardiography, nasal pressure transducer, oronasal thermistor, thoracic and abdominal respiratory effort belts, pulse oximetry, body position sensor, and microphone for snoring detection.^[20]^

Sleep stages and respiratory events were scored manually by experienced sleep technologists according to American Academy of Sleep Medicine scoring criteria version 2.6.^[21]^ Sleep stages were classified as wake (W), stage 1 (N1), stage 2 (N2), stage 3 (N3), and REM sleep. Total sleep time (TST) was defined as the sum of all sleep stages. Sleep efficiency was calculated as (TST/time in bed) × 100%.

Apnea was defined as a ≥ 90% reduction in airflow lasting ≥ 10 seconds. Hypopnea was defined as a ≥ 30% reduction in airflow lasting ≥ 10 seconds accompanied by either ≥ 3% oxygen desaturation or an electroencephalographic arousal. AHI was calculated as the total number of apneas plus hypopneas divided by TST in hours. In this cohort, hypopneas were scored using criteria incorporating both arousal and oxygen desaturation; consequently, the RDI and AHI were numerically equivalent throughout the dataset. AHI is therefore reported as the single frequency index throughout this manuscript, and RDI is not presented as a separate metric. The ODI was defined as the number of ≥ 3% oxygen desaturations per hour of sleep.

Duration parameters were meticulously quantified for each participant. Total AHD was calculated as the sum of all individual apnea and hypopnea event durations during the entire sleep period, expressed in minutes. Average apnea duration was calculated as the mean duration of all apnea events. Longest apnea duration was defined as the maximum duration of any single apnea event during the night, expressed in seconds. These duration metrics were computed separately for REM and non-REM sleep periods and for the total sleep period.

Oxygen saturation metrics included lowest oxygen saturation (nadir SpO_2_) during sleep, percentage of total sleep time with SpO_2_ < 90%, and percentage of total sleep time with SpO_2_ < 88%. REM sleep parameters included REM sleep duration (minutes), REM sleep percentage (REM sleep duration/TST × 100%), REM latency (time from sleep onset to first REM epoch), REM AHI (apneas plus hypopneas per hour of REM sleep), and REM-specific duration metrics. Computer-scored records were manually verified by experienced sleep physicians to ensure accuracy.

### 2.4. Laboratory measurements

Fasting venous blood samples were collected on the morning immediately following PSG, between 07:00 and 08:00 hours, after an overnight fast of at least 8 hours. Blood was drawn from an antecubital vein with minimal stasis and collected into serum separator tubes. Samples were allowed to clot for 30 minutes at room temperature, then centrifuged at 3000 rpm for 10 minutes. Serum aliquots were stored at −80°C until batch analysis to minimize inter-assay variability.

Serum prolactin concentrations were measured using electrochemiluminescence immunoassay on a Cobas e601 analyzer (Roche Diagnostics). The assay has a detection range of 0.047 to 470 ng/mL with an intra-assay coefficient of variation < 2.5% and an inter-assay coefficient of variation < 4.8%. Reference ranges are 4.0 to 15.2 ng/mL for men and 4.8 to 23.3 ng/mL for women.

TAS was determined using a commercially available colorimetric assay kit (Rel Assay Diagnostics) based on the decolorization of 2,2’-azinobis(3-ethylbenzothiazoline-6-sulfonic acid) radical cation by antioxidants present in the sample. The assay measures the combined activity of all antioxidants in serum. Results were expressed as mmol Trolox equivalent per liter (mmol Trolox Eq/L). The intra-assay and inter-assay coefficients of variation were < 3% and < 5%, respectively. All measurements were performed in duplicate according to manufacturer protocols, and samples were analyzed in a single batch by laboratory personnel blinded to clinical and polysomnographic data.

### 2.5. Statistical analysis

Statistical analyses were performed using SPSS version 25.0 (IBM Corporation). A two-tailed *P*-value < .05 was considered statistically significant for all analyses. Data distribution was assessed using the Shapiro–Wilk test. Continuous variables are presented as mean ± standard deviation for normally distributed data or median [interquartile range] for non-normally distributed data. Categorical variables are presented as frequency (percentage).

Baseline characteristics and polysomnographic parameters were compared across the 3 severity groups (control, mild/moderate OSA, and severe OSA) using one-way analysis of variance for normally distributed continuous variables, Kruskal–Wallis test for non-normally distributed continuous variables, and chi-square test or Fisher exact test (when expected cell frequencies < 5) for categorical variables. When overall tests indicated significant differences, pairwise comparisons were performed using independent samples *t*-test or Mann–Whitney *U* test as appropriate.

Correlations between biomarkers (prolactin and TAS) and polysomnographic parameters were assessed using Spearman rank correlation coefficients (ρ) due to non-normal distributions of several variables. Correlation strength was interpreted as weak (|ρ|<0.30), moderate (0.30≤|ρ|<0.50), or strong (|ρ|≥0.50). Correlation analyses were performed for the entire cohort to maximize statistical power and capture relationships across the full severity spectrum.^[22]^ To account for multiple correlation testing across the 6 polysomnographic parameters examined for each biomarker, *P*-values from Spearman correlation analyses were additionally adjusted using the Benjamini–Hochberg false discovery rate (FDR) procedure, with *q* < 0.05 considered significant after correction.

Sample size considerations: With n = 55 participants, we had 80% power to detect correlation coefficients of |ρ|≥0.36 at α = 0.05 (two-tailed). Post hoc power analysis was additionally performed using Fisher *z*-transformation to determine the minimum detectable correlation coefficient at 80% power for the study sample size; this indicated a minimum detectable |ρ| of 0.37. For 3-group comparisons with n = 12, 22, and 21, we had 80% power to detect effect sizes of *f* ≥ 0.46 (equivalent to η^2^ ≥ 0.17, representing large effects) at α = 0.05 using analysis of variance or Kruskal–Wallis tests.

Sensitivity analyses examined whether biomarker-PSG correlations differed by sex or BMI category (BMI < 30 vs ≥ 30 kg/m^2^) using Fisher *z*-transformation to test for significant differences between correlation coefficients. Missing data were minimal (<5% for any variable) and handled using complete case analysis. To formally test whether TAS and prolactin correlations with each polysomnographic parameter differed from one another, we additionally applied Steiger *Z* test for dependent (overlapping) correlations, which accounts for the correlation between the 2 biomarkers themselves.

## 3. Results

### 3.1. Participant characteristics

A total of 55 participants completed the study protocol and were included in the final analysis. Baseline demographic and clinical characteristics are presented in Table 1. The 3 groups (control, mild/moderate OSA, and severe OSA) did not differ significantly in age (median 42 [range 25–55] vs 45 [20–61] vs 47 [29–79] years, respectively; *P* = .42) or sex distribution (male sex: 42% vs 68% vs 86%, respectively; *P* = .15). BMI showed a nonsignificant trend toward higher values in the severe OSA group (31.4 ± 3.7 vs 29.1 ± 9.5 vs 31.4 ± 8.6 kg/m^2^, *P* = .08).

**Table 1:** Patient characteristics, polysomnographic parameters, and serum biomarker levels by OSA severity.

Variable	Control (n = 12)	Mild/Moderate OSA (n = 22)	Severe OSA (n = 21)	*P*-value
Age, yrs	42 [25–55]	45 [20–61]	47 [29–79]	.42
Male sex, n (%)	5 (42)	15 (68)	18 (86)	.15
Body mass index, kg/m^2^	31.4 ± 3.7	29.1 ± 9.5	31.4 ± 8.6	.08
Neck circumference, cm	36.2 ± 2.1	38.3 ± 4.2	41.8 ± 3.9*^,^**	<.001
Epworth Sleepiness Scale	7.2 ± 3.8	9.1 ± 4.6	11.3 ± 5.4	.12
AHI, events/h	1.9 [1.6–4.0]	10.4 [8.1–17.6]*	69.4 [39.2–82.1]*^,^**	<.001
ODI, events/h	0.3 [0.0–0.8]	2.7 [1.0–6.8]*	42.9 [25.6–64.3]*^,^**	<.001
Total apnea-hypopnea duration, min	4.5 [2.8–9.5]	23.0 [19.2–47.8]*	189 [121–231]*^,^**	<.001
Longest apnea duration, s	34 [29–43]	48 [37–58]*	70 [63–91]*^,^**	<.001
Lowest SpO2, %	91 [88–92]	86 [81–90]*	77 [66–82]*^,^**	<.001
REM sleep time, %	13.3 [9.1–16.4]	12.0 [6.5–15.8]	6.1 [4.3–11.6]*	.021
Prolactin, ng/mL	22.7 ± 9.8	14.7 ± 7.2*	12.7 ± 7.3*	.007
Total antioxidant status, mmol Trolox Eq/L	1.181 ± 0.068	1.122 ± 0.065*	1.111 ± 0.057*	.021

Data are presented as mean ± SD for normally distributed variables or median [interquartile range] for non-normally distributed variables. Categorical variables presented as n (%).

AHI = apnea-hypopnea index, ODI = Oxygen Desaturation Index, OSA = obstructive sleep apnea, REM = rapid eye movement, SpO_2_ = oxygen saturation.

* *P* < .05 versus Control group.

** *P* < .05 versus Mild/Moderate OSA group.

Neck circumference increased significantly with OSA severity (36.2 ± 2.1 vs 38.3 ± 4.2 vs 41.8 ± 3.9 cm; *P* < .001), with severe OSA patients demonstrating significantly larger neck circumference compared to both controls (*P* < .001) and mild/moderate OSA patients (*P* = .02). Epworth Sleepiness Scale scores showed a trend toward increased daytime sleepiness with greater OSA severity (7.2 ± 3.8 vs 9.1 ± 4.6 vs 11.3 ± 5.4, *P* = .12), though this did not reach statistical significance.

### 3.2. Polysomnographic parameters

Polysomnographic data are summarized in Table 1. As expected by design, AHI differed markedly across groups (control: 1.9 [1.6–4.0] events/h; mild/moderate OSA: 10.4 [8.1–17.6] events/h; severe OSA: 69.4 [39.2–82.1] events/h; *P* < .001). Similarly, the ODI increased progressively across severity groups (0.3 [0.0–0.8] vs 2.7 [1.0–6.8] vs 42.9 [25.6–64.3] events/h, *P* < .001).

Duration parameters demonstrated equally robust differences. Total AHD was markedly prolonged in severe OSA compared to both mild/moderate OSA and controls (189 [121–231] vs 23.0 [19.2–47.8] vs 4.5 [2.8–9.5] minutes, *P* < .001 for all pairwise comparisons). Longest apnea duration similarly increased across severity groups (34 [29–43] vs 48 [37–58] vs 70 [63–91] seconds; *P* < .001), with significant differences between all pairwise comparisons (control vs mild/moderate: *P* = .008; control vs severe: *P* < .001; and mild/moderate vs severe: *P* < .001).

Oxygen saturation metrics revealed severe hypoxemia in the severe OSA group. Lowest SpO_2_ (nadir SpO_2_) decreased progressively and significantly across all groups (control: 91% [88–92]; mild/moderate OSA: 86% [81–90]; severe OSA: 77% [66–82]; and overall *P* < .001). Pairwise comparisons demonstrated significant differences between controls and mild/moderate OSA (*P* = .013), controls and severe OSA (*P* < .001), and mild/moderate OSA and severe OSA (*P* < .001), indicating a stepwise deterioration in oxygenation across the severity spectrum (Fig. 3B).

**Figure 1.**
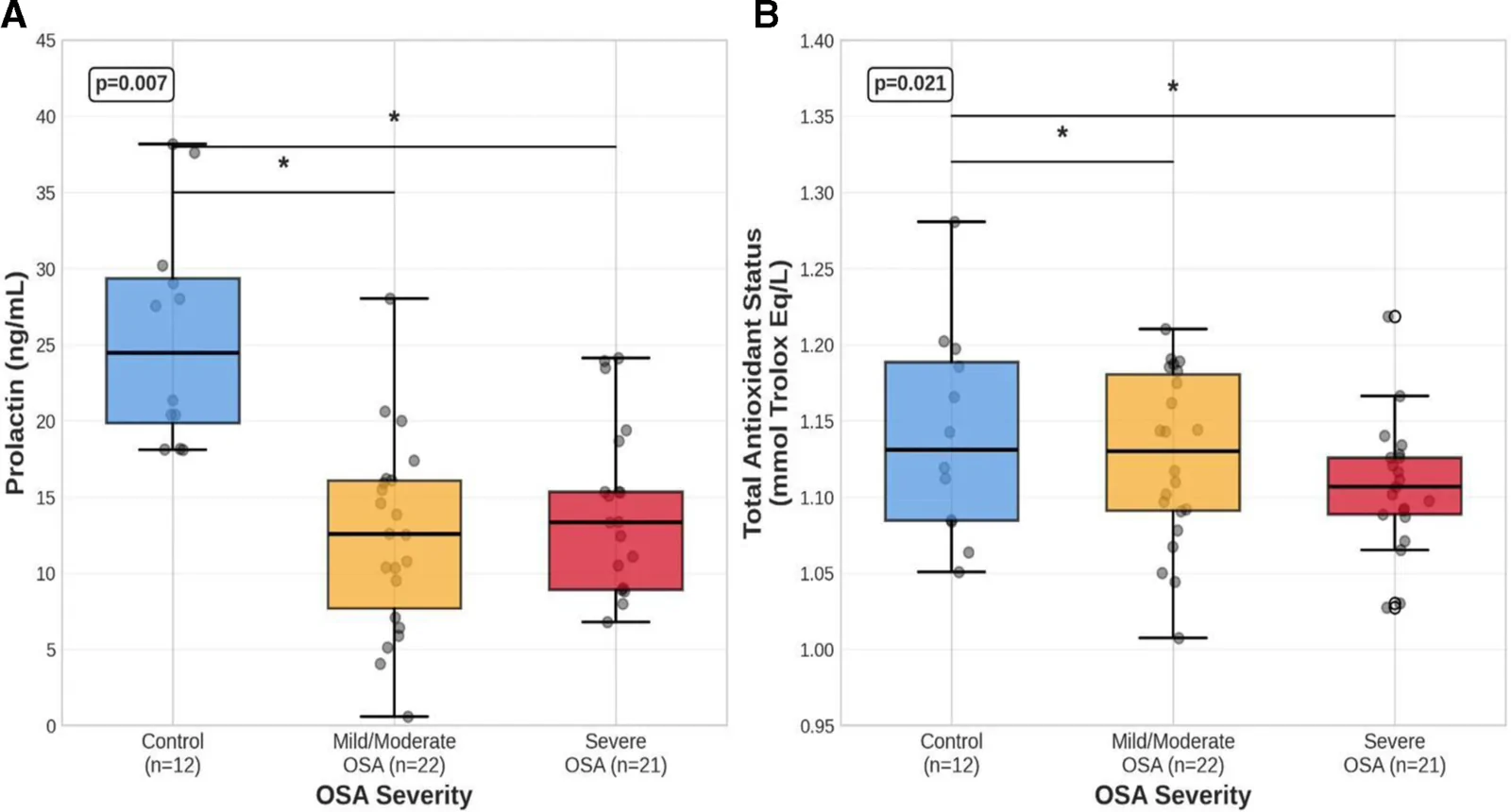
Serum prolactin and total antioxidant status levels across OSA severity groups. (A) Serum prolactin levels (ng/mL) demonstrated significant variation across OSA severity groups (Kruskal–Wallis test, *P* = .007). Control subjects (n = 12) showed markedly higher prolactin levels (median: 24.3 ng/mL, IQR: 20.1–29.5) compared to both mild/moderate OSA (n = 22; median: 12.5 ng/mL, IQR: 7.8–16.1) and severe OSA patients (n = 21; median: 13.8 ng/mL, IQR: 9.4–15.7). Post hoc pairwise comparisons revealed significant differences between controls and both OSA groups (*P < .05). (B) Total antioxidant status (TAS) expressed in mmol Trolox Equivalent/L showed significant differences among groups (Kruskal–Wallis test, *P* = .021). Controls demonstrated higher TAS values (median: 1.13 mmol Trolox Eq/L, IQR: 1.09–1.19) compared to severe OSA patients (median: 1.11 mmol Trolox Eq/L, IQR: 1.09–1.13), with mild/moderate OSA showing intermediate levels (median: 1.13 mmol Trolox Eq/L, IQR: 1.09–1.18). Post hoc testing revealed significant differences between controls and severe OSA (*P < .05). Box plots display median (central line), interquartile range (box), range (whiskers), and individual data points. IQR = interquartile range, OSA = obstructive sleep apnea.

**Figure 2.**
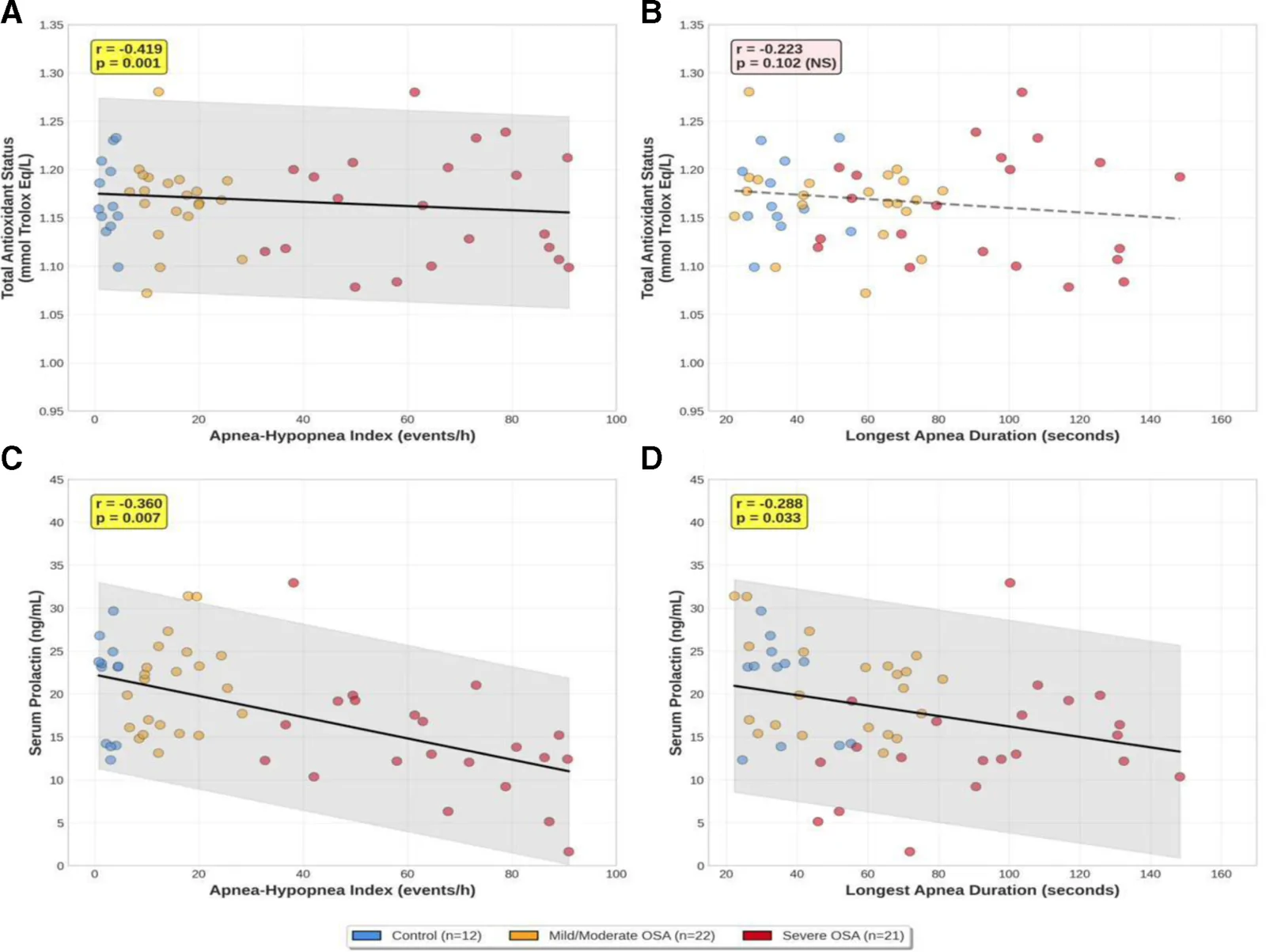
Differential correlation patterns between biomarkers and polysomnographic parameters. (A) Total antioxidant status (TAS) demonstrated a significant negative correlation with Apnea-Hypopnea Index (AHI; Spearman *r* = −0.419, *P* = .001), indicating that higher event frequency associates with lower antioxidant capacity. The regression line (black) with 95% confidence interval (gray shaded area) shows the strength of this association. (B) In contrast, TAS showed no significant correlation with longest apnea duration (Spearman *r* = −0.223, *P* = .102, NS), suggesting that antioxidant depletion relates to event frequency rather than individual event severity. (C) Serum prolactin exhibited a significant negative correlation with AHI (Spearman *r* = −0.360, *P* = .007), similar to TAS. (D) Prolactin also showed a significant negative correlation with longest apnea duration (Spearman *r* = −0.288, *P* = .033), unlike TAS. However, when directly compared using Steiger *Z* test for dependent correlations, the TAS and prolactin correlation coefficients did not differ significantly for any parameter shown here (all *P* ≥ .65; see Table 2 and Section 3.4); these descriptive patterns should therefore be interpreted as hypothesis-generating rather than confirmed evidence of dissociation. Data points are color-coded by OSA severity: blue = controls (n = 12), orange = mild/moderate OSA (n = 22), red = severe OSA (n = 21). NS = not significant; OSA = obstructive sleep apnea.

**Figure 3.**
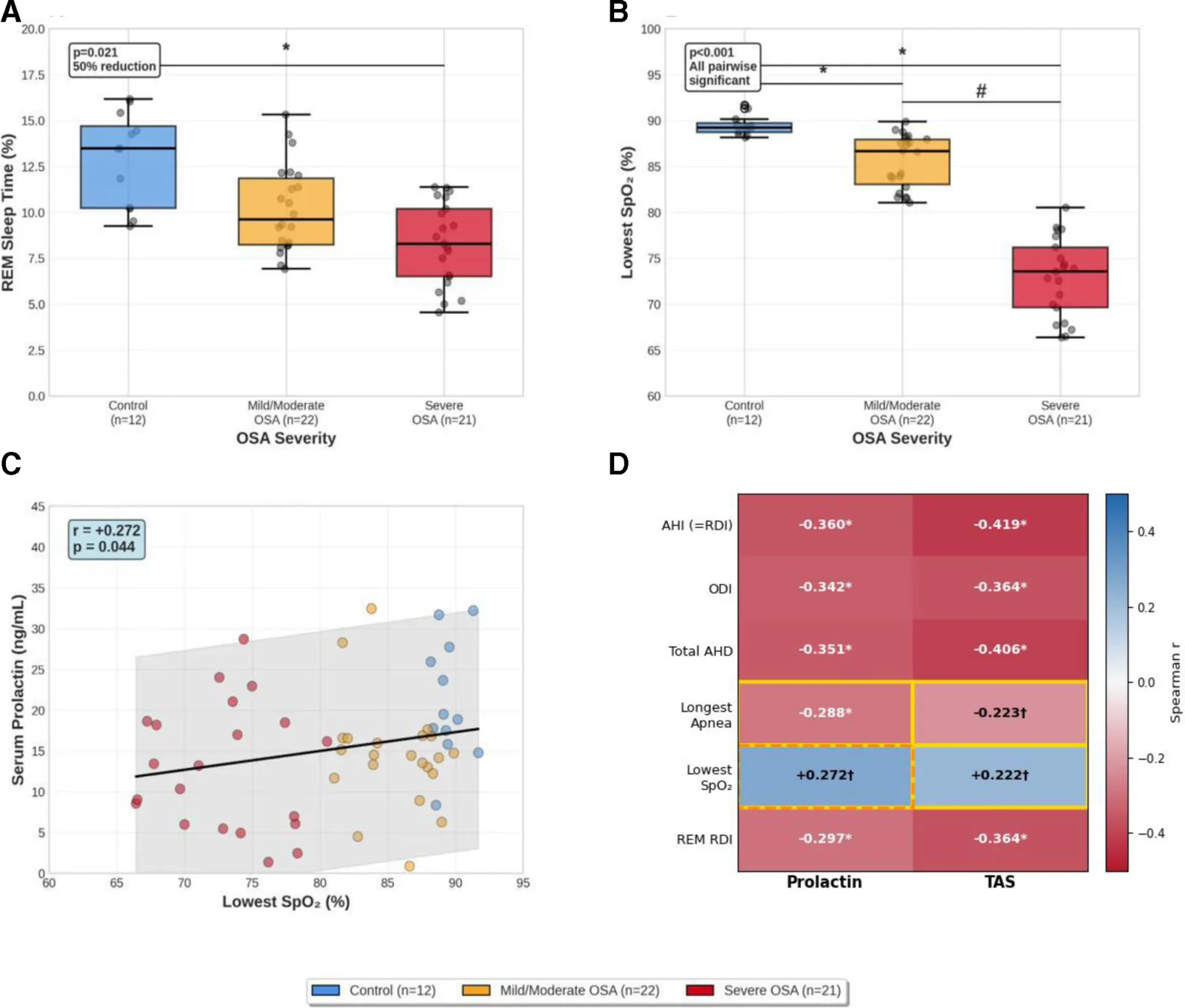
Sleep architecture, hypoxemia patterns, and comprehensive biomarker correlations. (A) REM sleep time as percentage of total sleep time showed progressive reduction with increasing OSA severity (Kruskal–Wallis test, *P* = .021). Controls (n = 12) demonstrated approximately 50% higher REM sleep percentage (median: 13.4%, IQR: 10.6–14.8) compared to severe OSA patients (n = 21; median: 8.7%, IQR: 6.8–10.3), with mild/moderate OSA (n = 22) showing intermediate values (median: 10.2%, IQR: 8.5–12.1). *P < .05 for controls vs severe OSA. (B) Lowest SpO_2_ during sleep declined markedly across severity groups (Kruskal–Wallis test, *P* < .001). All pairwise comparisons reached statistical significance: controls (median: 89.5%, IQR: 88.8–90.1) vs mild/moderate OSA (median: 85.2%, IQR: 83.5–87.3) vs severe OSA (median: 73.8%, IQR: 70.0–76.1). *P < .05 for all comparisons; #*P* < .001 for mild/moderate vs severe. (C) Serum prolactin demonstrated a positive correlation with Lowest SpO_2_ (Spearman *r*=+0.272, *P* = .044), suggesting that greater hypoxemia severity associates with hormonal dysregulation. (D) Correlation heatmap summarizing relationships between both biomarkers and key polysomnographic parameters (n = 55), revised to merge AHI and RDI – numerically equivalent in this cohort – into a single combined row, and to incorporate Benjamini–Hochberg FDR correction for multiple testing. Asterisks (*) denote correlations significant after FDR correction (*q* < 0.05); daggers (†) denote correlations significant at nominal *P* < .05 but not after correction, considered exploratory. Yellow borders highlight parameters (longest apnea duration and lowest SpO_2_) where TAS and prolactin crossed nominal significance thresholds differently; however, direct comparison of the 2 biomarkers’ correlation coefficients for these and all other parameters using Steiger *Z* test did not confirm a statistically significant difference (all *P* ≥ .65; Table 2), so these highlighted cells should be read as descriptive rather than as confirmed dissociation. The dashed orange border marks the prolactin–lowest SpO_2_ association, which did not retain significance after FDR correction (*q* = 0.053). Red indicates negative correlations; blue indicates positive correlations; color intensity reflects correlation strength. AHI = Apnea-Hypopnea Index, FDR = false discovery rate, IQR = interquartile range, ODI = Oxygen Desaturation Index, OSA = obstructive sleep apnea, REM = rapid eye movement, REM RDI = REM-specific Respiratory Disturbance Index, SpO_2_ = oxygen saturation, TAS = total antioxidant status, Total AHD = total apnea-hypopnea duration.

**Table 2:** Spearman correlation coefficients between biomarkers and polysomnographic parameters (revised: AHI and RDI rows merged as they were numerically equivalent in this cohort; FDR-adjusted *q*-values added per multiple-testing correction; Steiger *Z* test for dependent correlations added to formally compare TAS vs prolactin).

Parameter	Prolactin *r*	*P*	*q* (FDR)	TAS *r*	*P*	*q* (FDR)	Steiger P***
AHI (=RDI)*	−0.360	.007	0.017	−0.419	.001	0.013	.680
ODI	−0.342	.011	0.018	−0.364	.006	0.017	.879
Total apnea-hypopnea duration	−0.351	.009	0.017	−0.406	.002	0.013	.705
Longest apnea duration	−0.288	.033	0.044	−0.223	.102	0.103	.672
Lowest SpO_2_	+0.272	.044	0.053**	+0.222	.103	0.103	.744
REM respiratory disturbance index	−0.297	.028	0.041	−0.364	.006	0.017	.653

Values represent Spearman correlation coefficients (*r*), raw *P*-values, and Benjamini–Hochberg FDR-adjusted *q*-values (*q* < 0.05 considered significant after correction). N=55 for all correlations.

* AHI and the respiratory disturbance index (RDI) were numerically equivalent in this cohort (see Section 2.3) and are reported as a single combined row.

** This association was significant at nominal *P* < .05 but did not retain significance after FDR correction (*q* = 0.053) and is considered exploratory (see Sections 4.2 and 4.6).

*** Steiger *Z* test for dependent (overlapping) correlations, directly comparing the prolactin and TAS correlation coefficients for each parameter (accounting for *r* [TAS, prolactin] = 0.326); none reached significance (all *P* ≥ .65), indicating the apparent divergence between biomarkers was not formally confirmed (see Sections 3.4 and 4).

AHI = Apnea-Hypopnea Index, FDR = false discovery rate, ODI = Oxygen Desaturation Index, RDI = Respiratory Disturbance Index, REM = rapid eye movement, SpO_2_ = oxygen saturation, TAS = total antioxidant status.

REM sleep architecture showed marked disruption in severe OSA patients. REM sleep time as a percentage of total sleep time was reduced by approximately 50% in severe OSA compared to controls (Fig. 3A; 6.1% [4.3–11.6] vs 13.3% [9.1–16.4], *P* = .021), with the control versus severe OSA pairwise comparison reaching statistical significance (*P* = .007). REM sleep duration in minutes demonstrated a similar trend (23.5 [17.5–43.0] vs 48.5 [32.2–62.6] minutes, *P* = .095) that approached but did not reach conventional statistical significance.

### 3.3. Serum biomarker levels across OSA severity groups

Both serum prolactin and TAS decreased progressively with increasing OSA severity (Table 1). Serum prolactin concentrations differed significantly across the 3 groups (control: 22.7 ± 9.8 ng/mL; mild/moderate OSA: 14.7 ± 7.2 ng/mL; severe OSA: 12.7 ± 7.3 ng/mL; overall *P* = .007; Fig. 1A). Pairwise comparisons revealed significant decreases in both mild/moderate OSA (*P* = .014 vs controls) and severe OSA (*P* = .005 vs controls). However, the difference between mild/moderate and severe OSA groups did not reach statistical significance (*P* = .20), suggesting a threshold effect whereby prolactin suppression occurs early in OSA development but does not continue to decline proportionally with further severity increases.

TAS levels demonstrated a similar pattern of progressive decline (control: 1.181 ± 0.068 mmol Trolox Eq/L; mild/moderate OSA: 1.122 ± 0.065 mmol Trolox Eq/L; severe OSA: 1.111 ± 0.057 mmol Trolox Eq/L; overall *P* = .021; Fig. 1B). Both OSA groups showed significantly reduced TAS compared to controls (mild/moderate vs control: *P* = .034; severe vs control: *P* = .006). As with prolactin, the difference between mild/moderate and severe OSA did not achieve statistical significance (*P* = .56), suggesting that antioxidant depletion manifests early in OSA and may plateau rather than continuing to worsen linearly with AHI.

### 3.4. Differential correlation patterns: frequency versus duration parameters

Correlation analyses revealed descriptively different patterns for TAS versus prolactin in their relationships with polysomnographic parameters (Table 2, Fig. 3D), although direct statistical comparison of the 2 biomarkers’ correlations (reported below) did not confirm these differences as formally significant. These patterns constitute the primary observation of this study and are presented as a hypothesis-generating framework rather than confirmed evidence of distinct pathophysiological mechanisms.

TAS demonstrated significant negative correlations with event frequency parameters: AHI (=RDI in this cohort; *r* = −0.419, *P* = .001, *q* = 0.013) and ODI (*r* = −0.364, *P* = .006, *q* = 0.017). In contrast, TAS showed a moderate negative correlation with total AHD (*r* = −0.406, *P* = .002, *q* = 0.013) but crucially showed no significant correlation with longest apnea duration (*r* = −0.223, *P* = .102, *q* = 0.103) or lowest SpO_2_ (*r* = 0.222, *P* = .103, *q* = 0.103). This pattern is descriptively consistent with TAS depletion relating primarily to cumulative event burden (frequency and total duration) rather than individual event severity (longest duration or deepest desaturation; Fig. 2A–B).

Prolactin exhibited a descriptively different correlation profile. While prolactin also correlated negatively with frequency parameters (AHI [=RDI; *r* = −0.360, *P* = .007, *q* = 0.017]; ODI: *r* = −0.342, *P* = .011, *q* = 0.018), it additionally demonstrated significant negative correlations with duration parameters: total AHD (*r* = −0.351, *P* = .009, *q* = 0.017) and, critically, longest apnea duration (*r* = −0.288, *P* = .033, *q* = 0.044). Furthermore, prolactin showed a positive correlation with lowest SpO_2_ (*r* = 0.272, *P* = .044, *q* = 0.053; this specific association did not retain significance after correction for multiple testing and is considered exploratory, see Sections 4.2 and 4.6), indicating that lower prolactin levels associate with more severe hypoxemic nadirs (Fig. 3C). This multidimensional correlation pattern is descriptively consistent with prolactin disruption reflecting both event frequency and the severity of individual hypoxemic episodes (Fig. 2C–D).

To formally test whether the correlation patterns of TAS and prolactin differed from one another, rather than relying on informal comparison of significance thresholds, we compared the 2 biomarkers’ correlations with each polysomnographic parameter directly using Steiger *Z* test for dependent (overlapping) correlations, which accounts for the correlation between TAS and prolactin themselves (*r* = 0.326, *P* = .015). None of the 6 pairwise comparisons reached statistical significance (all |*Z*|≤0.45, *P* ≥ .65; Table 2), including for longest apnea duration (*Z* = 0.42, *P* = .672) and lowest SpO_2_ (*Z* = −0.33, *P* = .744) – the 2 parameters most central to our hypothesis of differential sensitivity. This indicates that, although TAS and prolactin crossed conventional significance thresholds differently for these parameters, their correlation coefficients were not formally distinguishable from one another in this sample.

To summarize: descriptively, TAS crossed the nominal significance threshold for frequency and cumulative duration metrics but not for individual event severity (longest apnea or lowest SpO_2_), whereas prolactin crossed this threshold for frequency, cumulative duration, and longest apnea duration, with a positive association with lowest SpO_2_ that was nominally but not FDR-significant. However, as noted above, direct comparison of the 2 biomarkers’ correlation coefficients did not confirm a statistically significant divergence for any parameter; these patterns should therefore be interpreted as descriptive and hypothesis-generating rather than as confirmed evidence of distinct mechanisms. These patterns are visualized in Figure 2.

### 3.5. REM sleep parameters and biomarker correlations

Both biomarkers demonstrated significant correlations with REM-specific respiratory parameters, though with different patterns of association strength. TAS showed a moderate negative correlation with the REM RDI (*r* = −0.364, *P* = .006, *q* = 0.017) and a weak-to-moderate correlation with the number of REM apneas (*r* = −0.310, *P* = .021). Prolactin correlated with the REM RDI (*r* = −0.297, *P* = .028, *q* = 0.041), the number of REM apneas (*r* = −0.304, *P* = .024), and REM duration in apnea (*r* = −0.302, *P* = .025).

These REM-specific correlations suggest that respiratory events occurring during REM sleep, which are typically longer and associated with greater oxygen desaturation than non-REM events, contribute meaningfully to both oxidative stress and neuroendocrine disruption. The observation that TAS shows its strongest REM correlation with event frequency (REM RDI) while prolactin correlates with both frequency and duration in REM sleep is descriptively consistent with, though does not independently confirm, the differential mechanistic interpretation discussed above.

### 3.6. Sensitivity analyses

Sensitivity analyses stratified by sex and BMI category revealed that the differential correlation patterns between TAS (frequency-dependent) and prolactin (frequency- and duration-sensitive) remained consistent across subgroups. Specifically, the TAS correlation with AHI did not differ significantly between men (*r* = −0.43) and women (*r* = −0.39, Fisher *z*-test *P* = .82), nor between participants with BMI < 30 kg/m^2^ (*r* = −0.45) versus ≥ 30 kg/m^2^ (*r* = −0.40, *P* = .76). Similarly, prolactin correlations with longest apnea duration did not differ by sex (men *r* = −0.31, women *r* = −0.24, *P* = .68) or BMI category (<30: *r* = −0.32, ≥30: *r* = −0.26, *P* = .71). These findings indicate the observed biomarker-PSG relationships are robust and not substantially confounded by sex or obesity status.

## 4. Discussion

This study demonstrates that serum prolactin and TAS exhibit differential correlation patterns with polysomnographic parameters in OSA, consistent with distinct pathophysiological mechanisms. While both biomarkers decreased progressively across OSA severity groups, TAS correlated primarily with event frequency metrics (AHI and ODI) but showed no significant relationship with longest apnea duration or lowest SpO_2_. In contrast, prolactin correlated with both frequency and duration parameters and showed a positive, nominally significant but not FDR-significant, correlation with lowest SpO_2_. Notably, when the TAS and prolactin correlation coefficients were compared directly for each parameter using Steiger *Z* test for dependent correlations, none of the differences reached statistical significance (Section 3.4; Table 2), indicating that these patterns, while descriptively distinct, have not been formally confirmed to differ between the 2 biomarkers and should be interpreted as hypothesis-generating. This differential pattern is descriptively consistent with the possibility that TAS reflects cumulative oxidative burden from repetitive respiratory events, whereas prolactin may indicate hypoxemic severity and neuroendocrine disruption from prolonged events. Both biomarkers associated with REM sleep parameters, consistent with REM sleep’s particular vulnerability to OSA-related pathophysiology. These findings suggest potential, hypothesis-generating utility for biomarker-based OSA phenotyping that would require prospective, adequately powered validation, including formal statistical comparison of biomarker correlations, beyond traditional AHI-based classification.

### 4.1. TAS and the frequency-dependent oxidative stress pathway

The frequency-dependent correlation pattern observed for TAS aligns with current understanding of oxidative stress pathophysiology in OSA. Each respiratory event represents an ischemia-reperfusion cycle: airway obstruction causes tissue hypoxia, followed by rapid reoxygenation upon airway reopening.^[9]^ This cyclical process activates NADPH oxidase, generates mitochondrial superoxide through electron transport chain dysfunction, and promotes xanthine oxidase-mediated reactive oxygen species production.^[23,24]^ The resulting oxidative burst depletes antioxidant reserves, including superoxide dismutase, catalase, and glutathione peroxidase.^[25]^

Our finding that TAS correlates with event frequency (AHI and ODI) but not with individual event duration (longest apnea) or severity (lowest SpO_2_) suggests oxidative stress accumulates based on the number of ischemia-reperfusion cycles rather than their individual intensity. This interpretation is supported by experimental models demonstrating that intermittent hypoxia induces greater oxidative stress than sustained hypoxia of equivalent total duration,^[26]^ and by studies showing oxidative biomarkers correlate more strongly with the frequency of desaturation events than with the desaturation nadir.^[27]^ From this perspective, each respiratory event contributes incrementally to oxidative burden, and cumulative damage depends primarily on total event count rather than whether individual events are brief or prolonged.

The moderate correlation between TAS and total AHD (*r* = −0.406) likely reflects the strong association between event frequency and cumulative duration – patients with more events naturally accumulate greater total duration. However, the absence of correlation with the longest apnea duration indicates that individual severe events do not disproportionately deplete antioxidants beyond their contribution to total burden. This pattern may help explain why some studies report inconsistent relationships between oxidative biomarkers and conventional severity metrics: if 2 patients have identical AHI but different average event durations, TAS would be similarly depleted despite potentially different clinical phenotypes.

### 4.2. Prolactin disruption: a multidimensional biomarker of hypoxemic severity

Prolactin demonstrated a somewhat different correlation profile, showing sensitivity to event frequency, duration, and (nominally) hypoxemic severity, although – as noted above – direct comparison with TAS did not confirm this difference as statistically significant. This multidimensional pattern may suggest prolactin disruption reflects neuroendocrine pathway alterations that respond to both repetitive sleep fragmentation and acute hypoxemic stress. Normal prolactin secretion follows a circadian pattern with nocturnal elevation beginning shortly after sleep onset and reaching peaks during slow-wave sleep.^[13,28]^ This sleep-associated secretion requires intact sleep architecture and appears mediated by reduced hypothalamic dopaminergic inhibition during sleep.^[14]^

OSA disrupts this delicate regulatory system through multiple mechanisms. Recurrent arousals fragment sleep architecture, reducing slow-wave sleep and thereby diminishing the physiological context for nocturnal prolactin elevation.^[29]^ Our finding of 50% REM sleep reduction in severe OSA patients, coupled with prolactin’s correlation with REM respiratory parameters, suggests sleep architecture disruption contributes to prolactin suppression. Additionally, repetitive hypoxemic events may activate stress pathways that suppress pituitary prolactin secretion – a hypothesis partially supported by (though not surviving correction for multiple testing; see below) our observation of a positive correlation between prolactin and lowest SpO_2_ (*r* = 0.272, *P* = .044, *q* = 0.053 after FDR correction). This exploratory finding suggests, but does not firmly establish, that patients experiencing more severe oxygen desaturation nadirs exhibit greater prolactin suppression, independent of event frequency.

Prolactin correlated with the longest apnea duration (*r* = −0.288, *P* = .033, *q* = 0.044), while TAS did not (*r* = −0.223, *P* = .102). However, Steiger *Z* test indicated this difference between the 2 biomarkers was not itself statistically significant (*Z* = 0.42, *P* = .672; Table 2), so it should be regarded as a descriptive rather than a confirmed contrast. This divergence may tentatively suggest that prolonged individual apneic events could exert specific effects on neuroendocrine regulation not fully captured by oxidative stress markers. Mechanistically, longer apneas produce more severe hypoxemia and hypercapnia, potentially triggering more intense sympathetic activation and stress hormone release that could suppress prolactin via hypothalamic pathways.^[30]^ The positive correlation with the lowest SpO_2_ (exploratory, as noted above, and likewise not formally distinguishable from the corresponding TAS correlation, *Z* = −0.33, *P* = .744) is broadly consistent with this interpretation: severe hypoxemic nadirs, typically associated with longer events, may suppress prolactin beyond the effects of event frequency alone.

Previous studies reporting variable prolactin findings in OSA^[15,16,31]^ may reflect a failure to account for this multidimensional relationship. Studies focusing solely on AHI would miss the duration and hypoxemic severity components, potentially explaining inconsistent results across cohorts with different event duration distributions. Our findings suggest that prolactin assessment might help identify patients at higher risk despite similar AHI values – specifically, those with longer individual events, although the deeper-desaturation association requires independent confirmation given that it did not survive multiple-testing correction.

### 4.3. REM sleep disruption and biomarker relationships

The marked REM sleep disruption observed in severe OSA (50% reduction in REM time percentage) and the significant correlations between both biomarkers and REM respiratory parameters warrant discussion. REM sleep demonstrates particular vulnerability to OSA through multiple mechanisms: physiological atonia of upper airway dilator muscles increases obstruction susceptibility, blunted ventilatory responses to hypoxia and hypercapnia prolong events, and a reduced arousal threshold may delay event termination.^[32,33]^

Both TAS and prolactin correlated with the REM RDI, though TAS showed a stronger correlation (*r* = −0.364 vs *r* = −0.297), suggesting that REM-related oxidative stress may be particularly pronounced. REM-predominant OSA has emerged as a clinically relevant phenotype associated with specific cardiovascular and metabolic consequences,^[34,35]^ and our findings suggest that oxidative stress and neuroendocrine disruption may contribute to these associations. The observation that prolactin correlated with REM apnea duration while maintaining the differential pattern (duration-sensitivity) seen in overall sleep parameters is consistent with the mechanistic interpretation that prolactin responds to both frequency and severity across all sleep stages.

### 4.4. Clinical implications and OSA phenotyping

The differential correlation patterns identified in this study have potential clinical implications, to be confirmed in future work. Current OSA diagnosis and management rely heavily on AHI, which quantifies only event frequency. However, accumulating evidence suggests that OSA comprises heterogeneous phenotypes with distinct pathophysiological mechanisms and clinical consequences.^[36,37]^ Our findings suggest that biomarker assessment could potentially complement AHI by capturing mechanistically relevant dimensions – specifically, cumulative oxidative burden (TAS) and hypoxemic severity with neuroendocrine disruption (prolactin).

Consider 2 hypothetical patients with identical AHI of 30 events/h. Patient A experiences frequent brief events with mild desaturation, while Patient B has fewer but longer events with severe desaturation. Traditional classification would assign both identical severity (moderate OSA). Our findings raise the speculative possibility that Patient A would demonstrate greater TAS depletion (higher event frequency), while Patient B would show more pronounced prolactin suppression (longer events and deeper desaturations), though this specific prediction has not been tested prospectively and, as noted above, the underlying biomarker-by-parameter differences were not statistically confirmed in direct comparison. This differential biomarker profile might eventually help identify patients requiring more tailored management despite similar AHI values but should be regarded as a hypothesis for future testing rather than a demonstrated clinical distinction.

The threshold effect observed for both biomarkers – significant decreases in mild/moderate OSA without further decline in severe OSA – suggests early intervention may be worth investigating further. If oxidative stress and neuroendocrine disruption manifest early and plateau, even mild-to-moderate OSA may initiate pathophysiological cascades warranting treatment. This interpretation aligns with emerging evidence that even mild OSA associates with cardiovascular consequences, though prospective studies correlating biomarker levels with clinical outcomes are needed to test this hypothesis.

### 4.5. Comparison with previous studies

Our TAS findings align with multiple previous studies reporting decreased antioxidant capacity in OSA.^[10–12]^ Our study additionally examines correlations with both frequency and duration parameters, suggesting that TAS associates specifically with event frequency in this cohort. This may help explain apparent inconsistencies in prior literature where some studies found strong AHI correlations while others did not – cohorts with varying duration distributions might show different correlation strengths if only AHI was examined.

Regarding prolactin, our findings of decreased levels in OSA align with some studies^[15,29]^ but differ from others reporting no change^[31]^ or even elevations in specific subgroups.^[32]^ The multidimensional correlation pattern we observed may help reconcile these discrepancies: studies that assessed only AHI would capture only 1 dimension of prolactin’s relationship with OSA. Furthermore, our exclusion of patients with conditions affecting prolactin (thyroid disease and medications) likely reduced confounding. The positive correlation between prolactin and lowest SpO_2_ – while not surviving correction for multiple testing in this sample – represents, to our knowledge, a novel and potentially hypothesis-generating observation warranting dedicated confirmation.

### 4.6. Limitations

Several limitations warrant consideration. First, the cross-sectional design precludes causal inference regarding whether biomarker alterations result from OSA or represent preexisting conditions that predispose to OSA. Longitudinal studies examining biomarker changes with CPAP therapy would strengthen causal interpretations. Second, sample size was modest, particularly for the control group (n = 12), limiting power for subgroup analyses. Effect sizes for the primary frequency-based findings were moderate-to-large and survived correction for multiple testing; sensitivity analyses also demonstrated broadly consistent patterns across sex and BMI strata.

Third, extensive exclusion criteria enhanced internal validity by minimizing confounding from conditions affecting oxidative stress and hormones but may limit generalizability to broader, real-world, clinic-based OSA populations with comorbidities. Fourth, we measured only TAS as an oxidative stress marker; assessment of specific antioxidants (superoxide dismutase, catalase, and glutathione) and oxidative damage markers (malondialdehyde and 8-isoprostane) would provide more comprehensive oxidative stress characterization. Similarly, measuring other hormones affected by sleep (cortisol, growth hormone, and testosterone) would clarify whether the prolactin pattern generalizes to other neuroendocrine axes.

Fifth, single-night PSG may not fully capture OSA severity given night-to-night variability. This limitation affects all PSG-based studies and argues for additional biomarker assessment as a complementary approach less susceptible to temporal variation. Sixth, although 11 of 12 biomarker-PSG correlations retained significance after Benjamini–Hochberg correction for multiple testing, the association between prolactin and lowest SpO2 did not (*q* = 0.053) and should be considered exploratory pending replication in an independent cohort. Post hoc power analysis indicated correlations with |ρ|<0.30 were detected with power below 65%, and the minimum correlation detectable at 80% power in this sample was |ρ|=0.37, underscoring that several specific associations require confirmation in larger samples. Seventh, and most importantly, the central premise of this study – that TAS and prolactin show differential (dissociated) correlation patterns – was evaluated informally by comparing which correlations crossed conventional significance thresholds. When the 2 biomarkers’ correlation coefficients were compared directly for each parameter using Steiger *Z* test for dependent correlations, none of the differences reached statistical significance (all *P* ≥ .65; Table 2, Section 3.4). This apparent divergence should therefore be regarded as descriptive and hypothesis-generating, not as confirmed evidence of distinct pathophysiological mechanisms; adequately powered studies with formal interaction or difference testing are needed before this framework is used for phenotyping or clinical decision-making. Finally, we did not assess cardiovascular or metabolic outcomes; future studies should examine whether biomarker-defined phenotypes predict differential clinical trajectories or treatment responses.

### 4.7. Future directions

Several research directions emerge from these findings. First, longitudinal studies should examine biomarker changes with CPAP therapy to help confirm OSA as the causative factor and determine whether both markers improve or show differential treatment responses. Second, larger cohorts with more granular OSA phenotyping (including hypoxic burden metrics, arousal indices, and sleep stage-specific parameters) could refine understanding of which specific PSG characteristics drive each biomarker’s alterations. Third, prospective outcome studies should assess whether biomarker profiles predict cardiovascular events, metabolic dysfunction, or neurocognitive decline independent of AHI. Fourth, interventional studies could examine whether antioxidant supplementation or other targeted therapies modify biomarker levels and clinical outcomes in biomarker-defined OSA phenotypes. Finally, the differential pattern concept should be tested with other biomarker pairs, and in larger, independent, clinic-based cohorts, to develop a more comprehensive mechanistic framework for OSA phenotyping.

## 5. Conclusions

This study provides preliminary, descriptive evidence that serum prolactin and TAS may show differential correlation patterns with polysomnographic parameters in OSA. However, direct statistical comparison of the 2 biomarkers’ correlations (Steiger *Z* test) did not confirm these differences as significant for any parameter tested (all *P* ≥ .65); the findings are therefore hypothesis-generating rather than confirmed evidence of distinct underlying pathophysiological mechanisms. TAS correlates primarily with event frequency metrics (AHI and ODI), a pattern descriptively consistent with cumulative oxidative burden from repetitive ischemia-reperfusion cycles. Prolactin showed sensitivity to event frequency and duration parameters (including longest apnea duration); its association with hypoxemic severity (lowest SpO_2_) was nominally significant but did not survive correction for multiple testing and should be treated as exploratory. Both biomarkers associate with REM sleep respiratory parameters, consistent with REM sleep’s particular vulnerability to OSA-related pathophysiology. The threshold effect observed for both biomarkers – significant decreases in mild/moderate OSA without further decline in severe OSA – suggests pathophysiological alterations may manifest early in disease development. Because the core dissociation hypothesis was not formally confirmed in this sample, these differential correlation patterns should be regarded as hypothesis-generating only, and any utility for biomarker-based OSA phenotyping remains speculative pending confirmation with formal statistical comparison in larger, independent cohorts, and would need to be demonstrated beyond traditional AHI-based classification. Given the modest sample size, the exploratory status of the lowest-SpO_2_ finding, and the nonsignificant direct comparison between biomarkers, prospective studies in larger, independent, clinic-based cohorts are needed to determine whether biomarker-defined phenotypes predict differential clinical outcomes and treatment responses.

## Acknowledgments

The authors thank all participants who contributed to this study. We are grateful to the sleep laboratory technicians at Bezmialem Vakif University Hospital for their technical assistance during polysomnography recordings.

## Author contributions

**Conceptualization:** Hamza Ogun, Esat Hayat, Fatmanur Okyaltirik.

**Data curation:** Hamza Ogun, Esat Hayat, Nuran Gokbulut.

**Formal analysis:** Hamza Ogun.

**Funding acquisition:** Hamza Ogun.

**Investigation:** Hamza Ogun, Esat Hayat, Nuran Gokbulut, Merve Yuzbasioglu, Deniz Kara.

**Methodology:** Hamza Ogun, Esat Hayat, Ömer Faruk Özer.

**Project administration:** Hamza Ogun.

**Resources:** Hamza Ogun, Ömer Faruk Özer.

**Software:** Hamza Ogun.

**Supervision:** Fatmanur Okyaltirik.

**Validation:** Hamza Ogun.

**Visualization:** Hamza Ogun.

**Writing – original draft:** Hamza Ogun, Ömer Faruk Özer.

**Writing – review & editing:** Hamza Ogun, Esat Hayat, Nuran Gokbulut, Merve Yuzbasioglu, Abdullah Kansu, Deniz Kara, Fatmanur Okyaltirik.
